# Psychosocial Vulnerabilities to Depression after Acute Coronary Syndrome: The Pivotal Role of Rumination in Predicting and Maintaining Depression

**DOI:** 10.3389/fpsyg.2012.00288

**Published:** 2012-08-13

**Authors:** Ellen-ge D. Denton, Nina Rieckmann, Karina W. Davidson, William F. Chaplin

**Affiliations:** ^1^Department of Medicine, Columbia University Medical CenterNew York, NY, USA; ^2^Berlin School of Public Health, Charité Universitätsmedizin BerlinBerlin, Germany; ^3^Department of Psychology, St. John’s UniversityJamaica, NY, USA

**Keywords:** psychosocial vulnerabilities, post-ACS, depression, rumination, cardiovascular disease

## Abstract

Psychosocial vulnerabilities may predispose individuals to develop depression after a significant life stressor, such as an acute coronary syndrome (ACS). The aims are (1) to examine the interrelations among vulnerabilities, and their relation with changes in depressive symptoms 3 months after ACS, (2) to prospectively assess whether rumination interacts with other vulnerabilities as a predictor of later depressive symptoms, and (3) to examine how these relations differ between post-ACS patients who meet diagnostic criteria for depression at baseline versus patients who do not. Within 1 week after hospitalization for ACS, and again after 3 months, 387 patients (41% female, 79.6% white, mean age 61) completed the Beck Depression Inventory (BDI) and measures of vulnerabilities (lack of pleasant events, dysfunctional attitudes, role transitions, poor dyadic adjustment). Exclusion criteria were a BDI score of 5–9, terminal illness, active substance abuse, cognitive impairment, and unavailability for follow-up visits. We used hierarchical regression modeling cross-sectionally and longitudinally. Controlling for baseline (in-hospital) depression and cardiovascular disease severity, vulnerabilities significantly predicted 3 month depression severity. Rumination independently predicted increased depression severity, above other vulnerabilities (β = 0.75, *p* < 0.001), and also interacted with poor dyadic adjustment (β = 0.32, *p* < 0.001) to amplify depression severity. Among initially non-depressed patients, the effects of vulnerabilities were amplified by rumination. In contrast, in patients who were already depressed at baseline, there was a direct effect of rumination above vulnerabilities on depression severity. Although all vulnerabilities predict depression 3 months after an ACS event has occurred rumination plays a key role to amplify the impact of vulnerabilities on depression among the initially non-depressed, and maintains depression among those who are already depressed.

## Introduction

In patients who have experienced an acute coronary syndrome (ACS), the risk of a major adverse cardiac event (MACE) recurrence and mortality is associated with depression (Carney et al., 2003; McGee et al., 2006; Lange and Herrmann-Lingen, 2007; Parker et al., 2008; Davidson et al., 2010; Denollet et al., 2010; Compare et al., 2011). Psychosocial vulnerabilities may predispose individuals to develop depression after a significant life stressor, such as an ACS. Previous studies have identified personality types, cognitive, interpersonal, and behavioral stressors that are related to elevated depressive symptoms after a cardiac event (Rieckmann et al., 2006; Denollet and Kupper, 2007; Gulliksson et al., 2007; Stafford et al., 2008). Grounded in the diathesis stress model, psychosocial vulnerabilities are thought to be stable person characteristics and social contexts that predispose persons to be more or less vulnerable to depression. Previous studies have identified behavioral (e.g., lack of pleasant events), interpersonal (e.g., lack of social support and attachments), and cognitive (e.g., maladaptive cognitions) vulnerabilities as correlates of depression symptoms among ACS patients (Contrada et al., 2008; Stafford et al., 2008). Rieckmann et al. (2006) showed that these vulnerabilities were independently associated with depressive status immediately after an ACS. Importantly, the vulnerabilities were only mildly to moderately correlated, and a substantial number of patients with elevated depressive symptoms had *no* psychosocial vulnerability, suggesting that the vulnerabilities were not merely the “products” of current depressive symptomatology. This finding has since been replicated using a cross-sectional design (Doyle et al., 2011). However, it is not clear whether vulnerabilities exacerbate depressive symptoms over time, or whether depressive symptoms in turn negatively impact vulnerabilities over time. Previous studies have considered the cross-sectional relations between psychosocial vulnerabilities and depression among cardiac patients (Rieckmann et al., 2006; Oxlad and Wade, 2008; Pedersen et al., 2008; Larsen and Christenfeld, 2009). However, longitudinal studies are lacking. Episodes of depression are typically relapsing/recurring and the cause of depression episodes is multifactorial (Naqvi et al., 2007; van Gestel et al., 2007). Likewise, psychosocial vulnerabilities vary and covary on a continuum throughout a lifetime (Harris, 2001; Birmaher et al., 2004; O’Sullivan, 2004; Hankin et al., 2009). The associations between psychosocial vulnerabilities and depression are not well understood for patients with cardiovascular disease and can be best understood with a prospective study.

Theories seeking to explain the role of rumination for patients with cardiovascular risk suggest that a “perseverative cognition” or rumination is associated with decreased parasympathetic activity and prolonged sympathetic activation, and thus may be the key vulnerability for explaining the underlying pathophysiology of depressive disorder and CVD risk (Brosschot et al., 2006). Nolen-Hoeksema defines rumination as the tendency to think repetitively and passively about negative emotions, focusing on symptoms of distress (Berkman et al., 2003; Brosschot et al., 2006). Ruminative responses to depressed mood involve repetitively focusing on the fact that one is depressed: on one’s symptoms of depression; and on the causes, meanings, and consequences of depressive symptoms. Moreover, rumination in response to first and/or lifetime major events is not uncommon (Garnefski et al., 2003; Enns and Cox, 2005; Grant and Beck, 2010; McIntosh et al., 2010). Rumination also impairs social relations and activities (Lam et al., 2003). Ciesla and Roberts (2002) found that rumination exacerbates cognitive factors to depression. We propose to test if rumination is an independent psychosocial vulnerability for depression, and/or an amplifier of other depression vulnerabilities, predicting future depression severity, in patients with cardiovascular disease.

Though studies have evaluated psychosocial predictors of depression in cardiac patients, no study has assessed rumination as a potential predictor of later depression among ACS patients. Additionally, there are no prospective studies assessing the impact of psychosocial depression vulnerabilities after an ACS on continuing depression. Therefore the aims of this research are to (1) evaluate the interplay between depression vulnerabilities, including rumination 3 months after an ACS, (2) to predict future depressive symptoms with vulnerabilities, rumination, and their interactions, when controlling for cross-sectional associations, and (3) to compare the associations of depression vulnerabilities and rumination with future depressive symptom severity in initially depressed and non-depressed Post-ACS patients.

We propose to examine the interplay between psychosocial vulnerability and depression for cardiovascular disease patients, using two different statistical approaches. We use cross-lagged correlation analysis and depressed group versus non-depressed group comparisons to investigate the complex associations that can occur between psychosocial vulnerability and depression disease onset and maintenance. Examination of cross-lagged paths, which contain both cross-sectional and longitudinal observational analyses, allows some possible causal pathways to be ruled out, and others to be further considered in future trials or interventions. For example, if elevated depressive symptoms only longitudinally predict rumination, when first controlling for their cross-sectional association, then future studies could determine if decreasing depression decreases rumination. However, if in this same analysis we discovered that rumination does not predict later increased depression (again, controlling for their cross-sectional analysis) then we need not design rumination interventions, hoping this would improve depression. We therefore use cross-lagged correlations to examine and rule out possible causal links among psychosocial vulnerabilities and depression. We contrast the possible casual pathways to depression between the initially depressed and non-depressed patient groups. In this subgroup analysis we can observe if trait-like vulnerabilities cause and/or exacerbate the depression severity for some individuals and not others. Thus, we target the individuals who are prone to specific psychosocial vulnerabilities that potentially play a causal role in their depression symptoms versus individuals whose psychosocial vulnerabilities may prevent them from recovery.

## Materials and Methods

The Coronary Psychosocial Evaluation Study (COPES) is a set of three studies, one of which is an observational cohort that included 457 patients; average age of 61 years old who had a recent ACS. The baseline analyses are based on all 457 patients. However, 55 patients died, were re-hospitalized, or could not be found at 3 month follow-up and 15 patients did not answer enough items to justify a regression-based approach to impute their total scores from the subset of answered items, thus, the sample size for the 3 month and longitudinal analyses is 387 (85% of the initial sample).

### Procedure

Patients were recruited on the coronary care and cardiac care step-down units of three hospitals in New York City, NY, USA, New Jersey, and New Haven, CT, USA, between May 2003 and March 2004. The institutional review boards of each hospital approved the study and informed consent was obtained from every patient. Patients were eligible if they met the criteria for ACS (either acute MI with or without ST-segment elevation or unstable angina with documented existing coronary artery disease) verified by the study cardiologists and had scores on the Beck Depression Inventory BDI-I (Beck et al., 1961) of either 0–4 (indicating minimal depressive symptoms), or ≥10 (indicating at least mild depressive symptoms), assessed within 1 week after the index ACS event. Exclusion criteria were terminal illness (life expectancy <1 year), cognitive impairment, substance abuse, and unavailability for follow-up.

After enrollment, a short medical exam was conducted, and patients completed questionnaires and interviews assessing psychosocial vulnerabilities within 7 days of the hospitalization. For Spanish-speaking patients, Spanish versions of the measures were used (see Rieckmann et al., 2006). Upon 3 month follow-up, patients were given a follow-up exam, the BDI-I, and again completed all measures of psychosocial vulnerabilities.

### Measures

#### Depressive symptoms

The 21 item BDI-I (Beck et al., 1961) was administered to all patients, as a measure of depression severity. Patients’ responses were measured using a 4 point scale (0–3). Scores were aggregated, with higher scores indicative of elevated depression symptom severity. The BDI had good internal consistency in the present sample (Cronbach alpha = 0.91).

#### Cognitive vulnerability

Using the 24 item Dysfunctional Attitude Scale [DAS-24] (Power et al., 1994), we assessed maladaptive cognitive schemata. The scale measures attitudes or beliefs that represent distorted cognitive styles (i.e., “if I fail partly, it is as bad as being a complete failure”). Responses ranged from 1 (totally disagree) to 7 (totally agree). Higher scores indicate higher levels of dysfunctional attitudes. The internal consistency of the DAS at baseline and 3 month follow-up was 0.83 and 0.85, respectively.

#### Behavioral vulnerability

The 20 item short version of the Pleasant Events Schedule for the Elderly (PES-E; Teri and Lewinsohn, 1982) was administered to assess the lack of pleasant events or behavioral vulnerability, according to patient report. Patients reported their frequency of engaging in 20 pleasant events. Scores were recoded so that higher mean scores on this scale represented increased *in*frequency of pleasant events. The internal consistency of the PES at baseline and 3 month follow-up was 0.82 and 0.84, respectively.

#### Interpersonal vulnerability

Interpersonal vulnerability was assessed using two different measures. The Role Transitions scale (Markowitz et al., 2000) listed six life changing events, and asked patients to indicate whether they had experienced any of these events within the past year. Items were summed to assess the total number of role transitions, with more role transitions indicative of increased interpersonal vulnerability. Role Transitions were not stable across time (as would be expected) with a test-re-test reliability across 3 months of 0.39 (*p* < 0.001).

Patients were also administered the 15 item Dyadic Adjustment Scale (Spanier, 1976), which was not comprised of multiple item responses. Patients were asked to name (1) one person closest to them and (2) to rate the extent they agree or disagree with them on 15 important issues (i.e., handling finances, household tasks, career decisions, etc.). Patient responses ranged from 1 (always disagree) to 6 (always agree). Scores were recoded so that higher scores represent higher disagreement (i.e., poorer dyadic adjustment). Test-re-test reliability was 0.55.

#### Rumination

The short ruminative responses scale (Treynor et al., 2003) was administered to each patient. This scale is comprised of two subscales: Reflection (five items) and Brooding (five items). Examples of Rumination Reflection include: “I analyze recent events to understand why I am depressed” or “I write down what I am thinking and analyze it” and Rumination Brooding: “I think, what am I doing to deserve this?” or “ I think, why can’t I handle things better?” Patients rated their ruminative behavior using a 4-point scale, (1) never to (4) always. Higher scores indicated more rumination. The internal consistency for rumination brooding at baseline was 0.84 and 0.87 at 3 months. The internal consistency for rumination reflection at baseline was 0.77 and 0.81 at 3 months.

### Covariates

#### Charlson comorbidity index

Patients were assessed using a weighted index of 22 medical conditions (Charlson et al., 1987). The score ranges from 0 to 37; the higher the score, the more the number of and severities of their co-morbid diseases.

#### Left ventricular ejection fraction

We used left ventricular ejection fraction (LVEF) as a measure of cardiac disease severity. Patients’ LVEF was obtained from cardiac diagnostic and intervention procedure reports (e.g., echocardiogram, left ventriculogram, Multiple Gated Acquisition Scan) performed during hospitalization. Values for LVEF were categorized into 4 groups: normal (=60), mild (45–59), moderate (30–44), and severe dysfunction (<30).

### Analyses

All predictor variables were centered. Cross-lagged correlations were used to address the cross-sectional and longitudinal associations between vulnerabilities and depression. We conducted 4 hierarchical regression analyses to identify the unique relations between each psychosocial vulnerability and depression severity, controlling for the other vulnerabilities. First, we analyzed the cross-sectional relations between 3-month psychosocial vulnerabilities and 3-month depression. Previous research with this data set had established the psychosocial vulnerability and depressive symptomology association immediately following ACS (Rieckmann et al., 2006). Thus our focus is to extend this model to 3-months and, most important consider these relations longitudinally. We analyzed the data longitudinally by regressing 3 month depression symptom severity, controlling for baseline depression severity, on the baseline psychosocial vulnerabilities, including rumination, and with rumination as a moderator for all other vulnerabilities. Finally, we considered the different impacts that psychosocial vulnerabilities and rumination may play in non-depressed and depressed patients. This allows for the differentiation between the role that psychosocial factors play in the maintenance of existing depressive symptoms, and the role they play in predicting the new onset of depressive symptoms.

In the hierarchical regression analyses, we used demographic covariates (age, sex, ethnicity, presence of partner, years of schooling, and work status), depression, medical comorbidity, and cardiac disease severity in the first step of the model. In the second step we used psychosocial vulnerabilities (dysfunctional attitudes, pleasant events, poor dyadic adjustment, and role transitions). Third, we used rumination, and finally the interaction terms (i.e., rumination × each respective vulnerability) in step four.

For the depressed and non-depressed subgroup analyses we determined the presence or absence of minor (*N* = 64)/major (*N* = 48) depression with the DISH interview (The Depression Interview and Structured Hamilton, Freedland et al., 2002). In addition, we classified patients who were prescribed an antidepressant (*N* = 57), as depressed. Thus, we compared 169 depressed patients and 288 non-depressed patients.

## Results

Table 1 presents the descriptive statistics for the demographic characteristics, covariates, and the variables used in the analyses. In general, depression vulnerabilities and depression severity decreased across the 3 months. And this decrease reached statistical significance for depression severity, dysfunctional attitudes, number of role transitions, and rumination brooding. Reflective rumination was unrelated to depression; thus, we only report results for the rumination subscale of “rumination brooding.”

**Table 1:** **Demographic and psychosocial vulnerabilities of 457 post-acute coronary syndrome patients at baseline and 387 post-acute coronary syndrome patients at 3 months**.

Variable	Baseline
(*N* = 457)	3 Months
(*N* = 387)	*p*-Value
Age, mean (SD), years	61.1 (12.47)		
Female%	41.4		
White%	79.6		
Hispanic%	10.7		
Currently living with partner%	62.1		
Currently employed%	49.9		
Education, mean (SD), years	13.65 (3.25)		
Charlson score, mean (SD)	1.37 (1.57)		
LVEF: normal ≥60%	45.1		
Depression severity (BDI), mean (SD)	8.96 (9.02)	6.88 (8.29)	<0.001
Dysfunctional attitudes (DAS), mean (SD)	78.91 (21.71)	75.39 (21.53)	<0.001
Pleasant events schedule (PES), mean (SD)	0.36 (0.28)	0.37 (0.27)	0.10
Poor dyadic adjustment (DYAD), mean (SD)	2.10 (0.77)	2.06 (0.77)	0.65
Number of role transitions (RLT), mean (SD)	0.86 (0.98)	0.72 (0.88)	0.01
Rumination brooding (RB), mean (SD)	9.76 (3.19)	9.46 (3.22)	0.01

*All displayed descriptive statistics are before any transformations were applied to the scales. Paired sampled *t*-tests were performed to test for significant mean change in scores*.

With increasing age, patients had lower scores on some of the vulnerabilities (Baseline: Rumination Brooding, *r* =  −0.21, *p* < 0.001; Role Transitions, *r* = −0.20, *p* < 0.001; Dyadic Adjustment Disagreement, *r* = −0.15, *p* = 0.002, 3 months: Rumination Brooding, *r* = −0.23, *p* < 0.001; Role Transitions, *r* = −0.10, *p* = 0.05; Dyadic Adjustment Disagreement, *r* = −0.15, *p* = 0.002).

Men exhibited significantly higher Dysfunctional Attitudes at baseline (Male = 82.10, SD = 20.74, Female = 74.38, SD = 22.30, *p* < 0.001) and 3 months (Male = 77.89, SD = 21.59, Female = 72.09, SD = 21.06, *p* = 0.01) than women. Women had significantly higher Rumination Brooding at 3 months; Male = 9.15, SD = 3.11, Female = 9.89, SD = 3.32, *p* = 0.02) when compared to men. Otherwise, there were no sex differences in any of the vulnerabilities.

**Figure 1:**
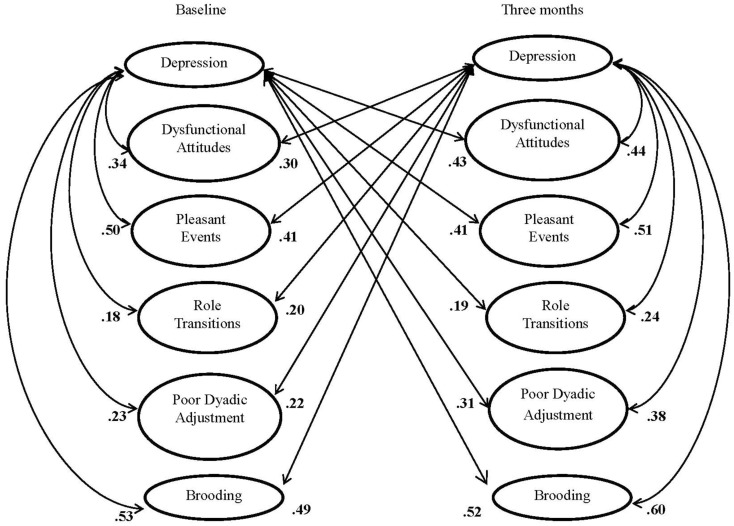
**Cross lagged correlations between depression, vulnerabilities, and rumination for baseline and 3 months**. All correlations are significant at **p < 0.001.

As seen in Table 2, the correlations among the psychosocial vulnerabilities were low to moderate cross-sectionally, both at baseline and at 3-months. Stability across the 3 months was highest for the Dysfunctional Attitudes and Rumination Brooding. In bivariate analyses, depression severity was moderately correlated with each of the vulnerabilities (Figure 1).

**Table 2:** **Cross-sectional and longitudinal intercorrelations among the psychosocial vulnerabilities and rumination**.

	Dysfunctional attitude	Pleasant events	Role transitions	Dyadic adjustment disagreement	Rumination brooding
Dysfunctional attitude	0.73**	0.26**	0.19**	0.17**	0.49**
Pleasant events	0.22**	0.65**	0.10*	0.26**	0.25**
Role transitions	0.06	0.06	0.39**	0.09	0.22**
Dyadic adjustment disagreement	0.10	0.17**	0.13**	0.55**	0.23**
Rumination brooding	0.38**	0.23**	0.16**	0.21**	0.74**

Correlates in the main diagonal are test-retest coefficients (baseline and 3 month correlates). Below and above the main diagonal are the baseline and 3 month intercorrelations, respectively. **p < 0.001.

### Cross-sectional model: Vulnerabilities assessed at 3 months as predictors of 3 months depression severity

After adjustment by covariates, including baseline depression severity, 3 month psychosocial vulnerabilities were concurrently related to 3 month depression severity. As shown in Table 3, together, all covariates predicted 48.2% of the variance in 3 month depression [*R* = 0.69, df = 9, *F*(9, 388) = 39.18, *p* < 0.001]. An additional 10.5% of 3 month depression severity was explained by the psychosocial vulnerabilities obtained at 3 months in block 2 [*R* = 0.77, *F*(13, 388) = 41.07, Δ*F* = 23.96, *p* < 0.001], and an additional 5.1% of 3 month depression severity was explained by rumination brooding at 3 months, in block 3 [*R* = 0.80, *F*(14, 388) = 47.11, Δ*F* = 54.41, *p* < 0.001]. Finally, an additional 4.1% of 3 month depression severity was explained by all of the, 3 month, rumination brooding and psychosocial vulnerability interactions (dysfunctional attitudes × rumination brooding; pleasant events infrequency × rumination brooding; role transitions × rumination brooding; and poor dyadic adjustment × rumination brooding), in block 4 [*R* = 0.82, *F*(18, 388) = 43.45, Δ*F* = 11.74, *p* < 0.001]. Interaction effects show that high rumination brooding served as a significant amplifier of infrequent pleasant events, (β = 0.57, *p* = 0.06), whereas low rumination brooding served to buffer the negative effect of poor dyadic adjustment (β = 0.32, *p* < 0.001), and dysfunctional attitudes (β = 0.01, *p* = 0.003 < 0.05), on 3 month depression severity. A fourth significant interaction effect between role transitions and rumination brooding indicates that depression severity is high if a person broods and if the person is undergoing role transitions (β = −0.19, *p* = 0.04). Depression severity is only low among patients who are neither undergoing role transitions nor brooding.

**Table 3:** **Hierarchical regression summary of the effect of psychosocial vulnerabilities at 3 months, rumination as an independent predictor and rumination as an amplifier on 3 month depression severity, post-ACS**.

	Δ*R*^2^	*B*	SE(*B*)	*p*-Value
Step 1	0.48**
Age		−0.05	0.03	0.07
Sex (1 = male; 2 = female)		−0.15	0.65	0.81
Partner (1 = yes; 2 = no)		1.16	0.67	0.8
Years of schooling		−0.28	0.10	0.01
Work status (1 = employed; 0 = unemployed)		0.29	0.73	0.69
Ethnicity (1 = Hispanic; 0 = non-Hispanic)		1.82	1.10	0.10
Baseline depression severity		0.57	0.04	<0.001
Charlson comorbidity score		0.09	0.22	0.68
LVEF		0.09	0.35	0.79
Step 2	0.11**
Dysfunctional attitudes		0.06	0.01	<0.001
Pleasant events scale		6.58	1.16	<0.001
Role transitions		0.82	0.32	0.01
Poor dyadic adjustment		1.58	0.39	<0.001
Step 3	0.05**
Rumination brooding		0.75	0.10	<0.001
Step 4	0.04**
DAS × brooding		0.01	0.003	0.003
PES × brooding		0.57	0.30	0.06
RLT × brooding		−0.19	0.10	0.04
DYAD × brooding		0.32	0.08	<0.001

DAS, dysfunctional attitudes; PES, pleasant events scale; DYAD, poor dyadic adjustment; RLT, number of role transitions. **p < 0.001.

### Longitudinal model: Baseline vulnerabilities as predictors of 3 months depression severity

**Figure 2:**
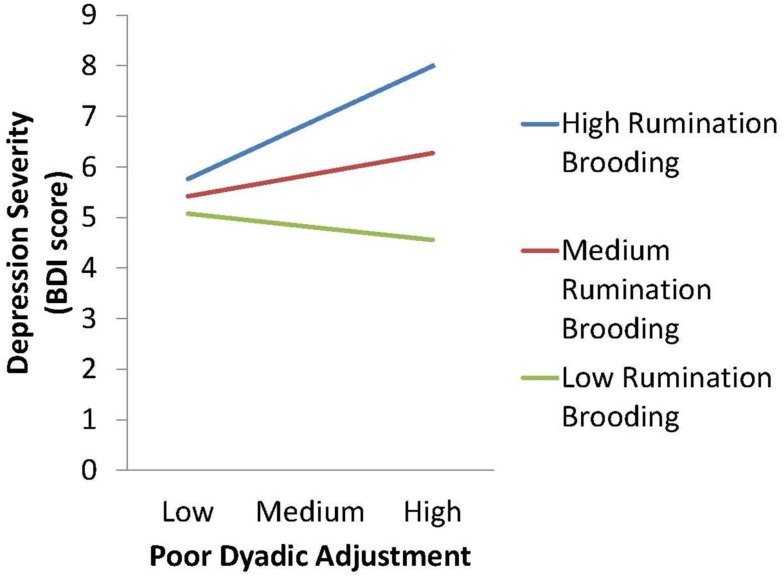
**Interaction effect of baseline poor dyadic adjustment and rumination brooding on 3 month depression severity, Post-ACS**.

After adjustment for covariates, including baseline depression and cardiovascular disease severity, baseline psychosocial vulnerabilities were longitudinally related to depression severity at 3 months. As shown in Table 4, the covariates predicted 48.5% of the variance in 3 month depression [*R* = 0.70, *F*(9, 375) = 38.35, *p* < 0.001], and an additional 1.6% of this depression severity was explained by baseline psychosocial vulnerabilities, in block 2 [*R* = 0.71, *F*(13, 375) = 27.96, Δ*F* = 2.85, *p* = 0.02], an additional 1.2% of 3 month depression severity was explained by rumination brooding, in block 3 [*R* = 0.72, *F*(14, 375) = 27.21, Δ*F* = 9.21, *p* = 0.003]. Finally an additional 1.4% of 3 month depression severity was explained by all of the baseline rumination brooding and psychosocial vulnerability interactions (dysfunctional attitudes × rumination brooding; pleasant events infrequency × rumination brooding; role transitions × rumination brooding; poor dyadic adjustment × rumination brooding), in block 4 [*R* = 0.73, *F*(18, 375) = 22.15, Δ*F* = 2.67, *p* = 0.03]. The interaction between baseline poor dyadic adjustment and rumination brooding independently predicted future, 3 month depression severity (*p* < 0.05; see Figure 2).

**Table 4:** **Hierarchical Regression summary of the effect of baseline psychosocial vulnerabilities, rumination as an independent predictor and rumination as an amplifier on 3 month depression severity, post-ACS**.

	Δ*R*^2^	*B*	SE(*B*)	*p*-Value
Step 1	0.48**
Age		−0.04	0.03	0.26
Sex (1 = male; 2 = female)		−0.43	0.66	0.52
Partner (1 = yes; 2 = no)		1.44	0.69	0.04
Years of schooling		−0.27	0.10	0.02
Work status (1 = employed; 2 = unemployed)		0.07	0.76	0.93
Ethnicity (1 = Hispanic; 2 = non-Hispanic)		1.74	1.12	0.12
Baseline depression severity		0.58	0.04	<0.001
Charlson comorbidity score		0.28	0.23	0.23
LVEF		0.26	0.34	0.45
Step 2	0.02*
Dysfunctional attitudes		0.03	0.02	0.07
Pleasant events scale		1.62	1.36	0.24
Role transitions		0.58	0.32	0.07
Poor dyadic adjustment		0.59	0.40	0.15
Step 3	0.01*
Rumination brooding		0.37	0.12	0.003
Step 4	0.01*
DAS × brooding		0.004	0.004	0.30
PES × brooding		0.24	0.39	0.54
RLT × brooding		−0.05	0.10	0.58
DYAD × brooding		0.27	0.10	0.01

DAS, dysfunctional attitudes; PES, pleasant events scale; DYAD, poor dyadic adjustment; RLT, number of role transitions. *p < 0.05, **p < 0.001.

### Sub-group differences between non-depressed and depressed at baseline

We used hierarchical regression to assess if the relation of baseline psychosocial vulnerabilities to 3 month depression severity was different for the depressed and non-depressed sub-groups. The overall moderating effect of depression status (depressed versus non-depressed subgroup) on the overall relation between baseline psychosocial vulnerabilities with 3 month depression was suggestive [*R* = 0.65, *F*(17, 395) = 16.02, Δ*F* = 2.15, *p* = 0.074]. Moreover, the 3-way interaction, of baseline rumination × psychosocial vulnerabilities × depressed versus non-depressed subgroup status to predict 3 month depression severity was significant [*R* = 0.69, *F*(15, 394) = 22.29, Δ*F* = 11.66, *p* = 0.001]. In exploring this effect we found that that high rumination amplified baseline vulnerabilities to depression severity for depressed patients but buffered depression severity for non-depressed patients.

We then applied the fully adjusted hierarchical model separately in the non-depressed patient sample and depressed patient sample. For depressed patients the demographic variables and covariates were significantly related to depression at 3 months [Step 1: *F*(9, 125) = 12.36, *p* < 0.001, *R*^2^ = 0.47], yet, the baseline psychosocial vulnerabilities were not significant predictors of 3 month depression severity [Step 2: Δ*F* = (4, 121) = 1.06, *p* = 0.38, Δ*R*^2^ = 0.02]. Baseline rumination brooding did directly predict 3 month depression severity for this group [Step 3: Δ*F* = (1, 120) = 6.65, *p* = 0.01, Δ*R*^2^ change = 0.03], but did not interact with baseline psychosocial vulnerabilities when predicting 3 month depression severity [Step 4: Δ*F* = (4, 116) = 0.275, *p* = 0.89, Δ*R*^2^ = 0.01].

A different pattern emerged for the non-depressed patients, the demographic variables and covariates were again significantly related to depression at 3 months [Step 1: *F*(9, 231) = 9.42, *p* < 0.001, *R*^2^ = 0.27], however, in contrast to the depressed subgroup, the baseline psychosocial vulnerabilities emerged as significant predictors of 3 month depression severity [Step 2: Δ*F* = (4, 227) = 3.71, *p* = 0.01, Δ*R*^2^ = 0.05]. Moreover, contrary to the results in the depressed subgroup, baseline rumination brooding did not significantly predict 3 month depression severity above baseline psychosocial vulnerabilities for the non-depressed sample [Step 3: Δ*F* = (1, 226) = 0.80, *p* = 0.37, Δ*R*^2^ change = 0.002], yet did interact with the baseline psychosocial vulnerabilities for non-depressed patients [Step 4: Δ*F* = (4, 222) = 5.31, *p* < 0.001, Δ*R*^2^ = 0.06] to exacerbate 3 month depression severity.

## Discussion

The purpose of this study was to extend our understanding of the relation among psychosocial depression vulnerabilities and depressive symptom severity in post-ACS patients, within a longitudinal data set. Results indicated that vulnerabilities are related to depression both cross-sectionally and longitudinally. Further, rumination brooding plays an important role as both a vulnerability and as an amplifier of other vulnerabilities for depressive symptoms after ACS. Overall, these findings replicate the results from previous cross-sectional designs that psychosocial vulnerabilities are independent correlates of depression severity among patients with cardiovascular disease (Rieckmann et al., 2006; Doyle et al., 2010). Full sample and sub-group findings are discussed in the following section. Finally, and most intriguingly vulnerabilities seem to act as precursors to depression in a non-depressed subsample at baseline and as maintaining factors for already depressed patients.

### Total sample: Psychosocial vulnerabilities and depression

Consistent with previous literature, these results show that 3 month psychosocial vulnerabilities are strong independent predictors of 3 month depression severity, controlling for the influence of cardiac disease severity, and initial depressive symptoms (Doyle et al., 2010). Psychosocial vulnerabilities explained a considerable amount of depression severity variance in the cross-sectional model illustrating the independent role of daily stressors on depression severity. Behavioral, cognitive, and interpersonal vulnerabilities had relatively weak intercorrelations, substantiating the unique variation among psychosocial vulnerabilities in this ACS sample. Cross-lagged correlations are interpreted to suggest two important findings regarding the relation between psychosocial vulnerabilities and depression. First, the lagged correlates suggest bidirectional and stable interrelations between psychosocial vulnerabilities and depression. Second, the differential strength of correlations at each time period suggest that the psychosocial vulnerabilities and depression influence is not strong at initial cardiac presentation (baseline cross-sectional correlations in Figure 1); however, this influence intensifies over time (3 month cross-sectional correlations in Figure 1).

### Rumination

In the total sample, we found higher rumination brooding to significantly predict future depression severity independent of the traditional psychosocial vulnerabilities. Ciesla and Roberts (2007) hypothesized rumination to be a “ non-specific vulnerability” to depression, and the cardiovascular literature also identifies rumination as a risk factor to poor health outcome, explaining physiological mechanisms between depression symptoms and cardiovascular health (Brosschot et al., 2006; Larsen and Christenfeld, 2009). Also of interest is that reflective rumination was not a psychosocial vulnerability for depression. Our findings suggest that patients who reflect are at no increased risk of elevated depression symptoms.

### Rumination amplifies depression severity

In our total sample, rumination exacerbates depression severity when combined with cognitive, behavioral, and interpersonal stressors. The interaction effect of rumination brooding and poor dyadic adjustment (interpersonal vulnerability) independently predicted higher depression severity at 3 months. This finding demonstrates the complexity, yet importance, of close relationships and future mental health. It also supports the moderation model, coined by Ciesla and Roberts (2007). The moderation model explains that the relation between rumination and depression is conditional (Ciesla and Roberts, 2007). These authors found that level of negative cognition (cognitive vulnerability) moderated the relation between rumination and depression. Our findings extend the moderation model. In our sample, rumination brooding moderated the effect of dyadic adjustment so that high partner disagreement had no effect on depression severity at 3 months when there was no rumination brooding, with moderate rumination brooding, high levels of partner disagreement were moderately associated with depressive symptoms, and partner disagreement had the worst effect in the presence of high levels of rumination brooding.

### Subgroup analyses: Psychosocial vulnerabilities and depression

Perhaps the most intriguing finding of this study is that patterns of psychosocial vulnerability influence differed by initial depression group status post-ACS. Specifically, cognitive, behavioral, and interpersonal vulnerabilities appear to influence future depression symptoms in non-depressed patients, whereas rumination brooding significantly influenced future depression symptoms only among depressed patients. Rumination brooding appears to be a mechanism for *maintaining depression* for post-ACS patients who have elevated depressive symptoms at baseline. For those post-ACS patients who are not depressed, the standard psychosocial vulnerabilities – cognitive, behavioral, and interpersonal – are the ones that appear to predispose them to become depressed in the future. Thus, prevention might focus on these factors.

### Rumination

Although we generally found (in the full sample) that rumination brooding was a unique and independent predictor of 3 month depression severity, subgroup analyses suggested a more complex process. Baseline rumination brooding independently predicted future depression severity for depressed patients, but not for non-depressed patients. Nolen-Hoeksema posits that ruminative responses prolong and intensify depressive episodes, whereas distraction from rumination response can serve as intervention, shortening, and diminishing depressive episodes (Nolen-Hoeksema, 1991; Enns and Cox, 2005). Further, the maintenance of physiological arousal for depressed cardiovascular disease patients is hypothesized to place these patients at increased risk of disease recurrence and/or mortality (Brosschot et al., 2006). We interpret our findings to indicate that rumination brooding maintains depression symptoms for patients who are already depressed. We can then speculate that clinical interventions to reduce rumination brooding symptoms can alleviate depression symptoms in those already depressed. We also found that baseline rumination brooding amplifies other psychosocial vulnerabilities in predicting future depression severity uniquely for initially non-depressed CVD patients. Thus, our study replicates the indirect and predictive effect of rumination on depression severity (Ciesla and Roberts, 2002; Ciesla and Roberts, 2007; Robinson and Alloy, 2003).

### Limitations

One limitation of this study is that our participants were identified post-ACS, thus prior history of depression, depression vulnerabilities, and ruminative cognitions are not known. The interpretation of these results is in the context of an important 3 month window, or “snapshot,” following an ACS event. Also, this study population may not be representative of all ACS patients, due to the exclusion of patients with BDI scores 5–9. However, this study design captures patients who are at elevated risk for ACS recurrence and mortality (those with BDI score ≥ 10) and demonstrates the complex interplay that depression vulnerabilities have on their recovery prognosis. Our findings are based on a vital Post-ACS recovery period and the complex role that rumination plays during this time underscores the importance of further research on this construct. Women had higher rumination brooding scores relative to men. Men had higher dysfunctional attitude scores (cognitive vulnerability) relative to women. The sex differences have theoretical and practical implications. Theoretically, gender may be a moderating determinant of depression severity. Practically, it is possible that cardiac treatment and prevention efforts can target women with increased psychosocial vulnerability and thereby reduce the risk of relatively worse recovery for women (Gulliksson et al., 2007). For men, cognitive-based interventions may be most therapeutic (Compare et al., 2011). Although we observed these gender differences in our patient sample, gender differences were not included in the overall model predicting depression severity.

## Conclusion

The current findings suggest that cognitive, behavioral, interpersonal vulnerabilities, and rumination brooding are longitudinally associated with depression severity after an ACS. As depressive symptoms are related to the effects of rumination brooding and psychosocial vulnerabilities, CHD recovery, and treatment interventions should consider social and trait-like influences on patients’ health outcome. Rumination brooding uniquely contributes as an independent and amplifying psychosocial vulnerability of depression severity. Thus it can be considered a clinical marker of 3 month depression in post-ACS patients. Based on these findings, treatment approaches for ACS patients should consider psychosocial vulnerabilities as possible contributors to CVD health outcomes.

## Conflict of Interest Statement

The authors declare that the research was conducted in the absence of any commercial or financial relationships that could be construed as a potential conflict of interest.
